# Feasibility study of a menstrual health behaviour change intervention for women and girls with intellectual disabilities and their caregivers for Vanuatu’s humanitarian responses

**DOI:** 10.1371/journal.pgph.0002244

**Published:** 2024-01-19

**Authors:** Jane Wilbur, Casey-Lynn Crow, Relvie Poilapa, Chloe Morrison

**Affiliations:** 1 International Centre for Evidence in Disability (ICED), London School of Hygiene & Tropical Medicine, London, United Kingdom; 2 World Vision Vanuatu, Port Vila, Vanuatu; University of Montreal School of Public Health: Universite de Montreal Ecole de Sante Publique, CANADA

## Abstract

The Veivanua campaign is a menstrual health intervention for people with intellectual disabilities and their caregivers in Vanuatu’s humanitarian setting. The campaign was adapted from the Bishesta campaign delivered in Nepal’s development setting. This feasibility study is designed to assess the feasibility and acceptability of the Veivanua campaign to understand if efficacy testing is warranted. The Veivanua campaign was delivered to a preselected group of 30 young people (individuals with intellectual disabilities) and 35 caregivers (males and females). Data were collected through several qualitative tools to allow for methods triangulation: process monitoring, post-intervention in-depth interviews with caregivers and nine young people, observation of young persons, photovoice and ranking with two young people, campaign resource ranking, and key informant interviews with staff involved in the intervention. Data were analysed thematically using Nvivo 12. Results show that the Veivanua campaign is feasible. Male and female caregivers reported an increased ability to support young people’s menstrual health and greater preparedness for the next emergency. Young people understood the training and applied their learning. Key informants want to scale up the intervention in their humanitarian responses. Several changes were made to the adapted campaign, but similar outcomes were recorded in Nepal and Vanuatu. All target behaviours improved, and campaign resources were used, but many caregivers found the menstrual calendar confusing. The intervention was not delivered with fidelity but responded to the context. The campaign cost more than the Bishesta campaign because procurement was more expensive in Vanuatu. In conclusion, this is the first intervention globally, so it begins to fill a substantial gap, but more must be done. As the Veivanua campaign is feasible, it requires efficacy testing in Vanuatu. It should also be adapted to humanitarian crises in other countries to support the menstrual health of this previously excluded population.

## Introduction

### Background

Achieving menstrual health means accessing relevant information about the menstrual cycle and hygiene behaviours, affordable and effective menstrual materials, and accessible water, sanitation, and hygiene (WASH) facilities where people can change and dispose of their menstrual materials and wash their bodies privately. It also includes access to medical assistance for any menstrual-related discomforts and disorders and an environment free from stigma and discrimination [1]. Menstrual health is vital for gender equality, sexual and reproductive health, and participation in education and employment [2–6]. Yet, many women and girls in low- and middle-income countries have not achieved menstrual health. Consequently, it must be integral to poverty reduction efforts, including during humanitarian crises [7].

Women and girls with disabilities face numerous challenges to achieving menstrual health, which can be exacerbated in humanitarian crises [8–15]. Barriers include exclusion from menstrual health policies and practices, inaccessible WASH facilities, and a lack of support and guidance for caregivers. People with intellectual disabilities face extreme outcomes from inadequate menstrual health, including physical restraint, abuse and sterilisation [16, 17]. Evidence reveals that the sexual and reproductive health (SRH) of persons with disabilities is acutely neglected [18–20]. For instance, Hameed et al’s. recent systematic review of efforts to promote SRH (which includes menstrual health) for persons with disabilities found that only 5% of the 400 studies included centred on interventions, most of which were delivered in upper-middle-income countries and urban settings, predominately focused on information provision and very few evaluated the intervention’s effectiveness [18]. The authors called for the trialling and evaluating of SRH interventions in low-middle-income settings and those that address barriers people with disabilities face in accessing SRH interventions [18]. Arguably, SRH for persons with disabilities is overlooked because of disability discrimination, including the misconception that persons with disabilities are asexual and that they do not have the same reproductive systems as those without disabilities, meaning they do not menstruate and cannot have children [17, 18, 21–23].

To address this, the Bishesta campaign–a menstrual health behaviour change intervention for people with intellectual disabilities and their caregivers–was developed, delivered and evaluated in Nepal [24]. Evaluation findings show that the Bishesta campaign was feasible and acceptable to the target groups and facilitators, improvements were recorded across all target behaviours, and there were indications that the young people with intellectual disabilities felt more autonomous and confident when menstruating after the training. As the Bishesta campaign was successful on a small scale, it needed efficacy testing before scaling up or adapting for a different setting. Information on the evidence generated through the study and details about the Bishesta campaign’s design, delivery, and evaluation are published elsewhere [12, 16, 17, 24–27].

### Adapting the Bishesta campaign for humanitarian responses in Vanuatu

The ‘Shifting Humanitarian Norms’ research, conducted by the LSHTM and World Vision, aimed to adapt the Bishesta campaign for humanitarian responses in Vanuatu and test its feasibility. The research applies the Behaviour Centred Design, which employs a theory of change, behavioural determinants and a programme design procedure [28]. The latter applies five steps to design a behaviour change intervention: Assess, Build, Create, Deliver and Evaluate.

In the Shifting Humanitarian Norms research, a literature review about menstrual health for women and girls with and without disabilities in humanitarian responses was completed within the Assess step [14]. It revealed a significant gap in evidence related to achieving menstrual health for this population in humanitarian responses compared to women and girls without disabilities [14].

Within the Build step, formative research was conducted in Vanuatu to further explore the menstrual health experiences of women and girls with intellectual disabilities and their caregivers during humanitarian emergencies in the country. It found that the menstrual health requirements of this population are not considered effectively, which could exclude them from menstrual health interventions [15]. The study also found that men and women support girls with intellectual disabilities when menstruating, so male caregivers must be included in interventions. Within a context of high gender-based violence, families with women and girls with intellectual disabilities are particularly concerned about finding safe and private places to evacuate during an emergency. Due to trauma, young people’s support needs may increase, making it even harder for caregivers to work and rebuild after crises. Hygiene kits that include menstrual materials are valued by families, especially as they are usually unaffordable. However, more materials are required, and a greater choice of products to meet diverse needs, such as incontinence. Distribution centres must be more accessible and coupled with house-to-house delivery to support those unable to leave home easily. Finally, study results highlighted that menstrual health interventions for people with and without disabilities are important to public health efforts before, during, and after humanitarian crises.

In the Create step, the Bishesta campaign was adapted for Vanuatu’s humanitarian setting based on the evidence generated. The Veivanua campaign targets young people with intellectual disabilities aged 15–31 and their caregivers living in the SANMA province. Both groups have three target behaviours. Young person: use a menstrual material, use pain relief, and do not show used menstrual materials in public. Caregiver: provide enough menstrual materials, provide pain relief, and show love and emotional support.

The campaign focuses on two fictitious characters: Veivanua (meaning a girl or woman who belongs to this place), a young woman with intellectual disabilities, and her caregiver Votahenavanua (meaning a woman who inspires others and takes care of the needs of others in her community, island or nation). Both practice the target behaviours and feel comfortable and confident when Veivanua is menstruating. Veivanua’s father also supports her in managing her menstruation.

The intervention included ‘period packs’ for young women containing a menstrual storage bag, reusable menstrual materials, a small drawstring bag, a menstrual bin, a Veivanua doll and a visual story. The latter depicts Veivanua menstruating for the first time, her father and Votahenavanua supporting her to understand menstruation and how to manage it hygienically and with dignity, preparing for an emergency and taking the menstrual storage bag with reusable menstrual materials in it whilst evacuating. Caregivers were given a menstrual calendar to track the young person’s menstrual cycle.

A Veivanua doll, with removable clothes and underwear, a clean and used menstrual materials were used in the training to help young people understand how and when to change a menstrual material, how to dispose or wash and dry it, and where they may experience menstrual discomforts and how to relieve them. A small Veivanua doll was offered to each young person so they could practice the target behaviours on it when they themselves were not menstruating.

For the Deliver step, the Veivanua campaign was implemented by four World Vision staff, including a woman with disabilities (called ‘facilitators’ in this article), under the direction and guidance of another World Vision staff member and the LSHTM. Following detailed guidance, the facilitators delivered the campaign in group settings and people’s homes. The Bishesta and Veivanua campaigns sought to encourage young people’s autonomy, confidence and agency when menstruating. Examples include encouraging young people to collect menstrual materials from the storage bag and dispose of used materials independently instead of relying on caregivers; facilitators also encouraged caregivers to allow young people time to answer questions directed at them instead of answering themselves, and facilitators always reacted positively to responses from young people to build their confidence. Staff visited people’s homes to monitor the adoption of target behaviours and answer any questions participants had. The process of implementing the campaign was also monitored.

This study aims to assess the feasibility and acceptability of the Veivanua campaign in the SANMA province, Vanuatu, by investigating its acceptability, demand, implementation, practicality and adaptation [29]. This study sits within the Evaluate step of the Behaviour Centred Design.

### Terminology

In this article, we refer to ‘women and girls’ to increase readability, yet the authors recognise that menstrual health is relevant to all people who menstruate, irrespective of gender identity and that not all women and girls menstruate.

## Materials and methods

### Research design

This is a feasibility study designed to assess if a future randomised control trial is warranted [30]. The rationale for completing a feasibility study draws on Bowen et al., who state that such research can be undertaken when ‘previous interventions had positive outcomes but in different settings than the one of interest’ (p3) [29]. This study applies Bowen et al.’s feasibility study framework [29]. This systematically guides the evaluation of an intervention by assessing eight focus areas: acceptability, demand, implementation, practicality, adaptation, integration, expansion, and limited-efficacy testing. In this study, the first five focus areas were considered. Table 1 presents these focus areas with the corresponding Veivanua feasibility study outcome indicators. The final two focus areas within the feasibility study framework, expansion and limited-efficacy testing, were outside the boundaries of this research and should be assessed in future studies. Process monitoring data were gathered during the delivery of the intervention to assess if a) the campaign was implemented as intended, b) how many participants received every training session and how frequently they were exposed to the campaign, and c) the degree to which the participants used the materials in the period packs.

**Table 1:** Bowen’s feasibility study framework and the Veivanua study outcome indicators.

Bowen et al.’s feasibility study framework			Veivanua study	
Focus area	Topics to investigate	Outcomes of interest	Outcome indicators	Data collection methods
**Acceptability**	How the participants and implementers react to the intervention	Satisfaction	Participants reporting that they positively benefited from the campaign	In-depth interview, photovoice, observation, field notes
			Participants reporting satisfaction with the resources provided in the Veivanua campaign	
			Participants reporting that they feel more prepared for another emergency	
		Intent to continue use		
		Perceived appropriateness		
		Fit within organisation culture	Facilitators reporting positively about the campaign	Key informant interview
			World Vision expressing intent to continue implementation and/or integrate it into their wider menstrual health interventions during emergencies	
		Perceived positive or negative effects on organisation	Facilitators indicating that they want to continue delivering the campaign, including during emergencies	
**Demand**	Estimated or actual use of intervention activities in a defined target group	Actual use of period pack contents	Young people using the period pack contents	Process monitoring tools, in-depth interview, photovoice
			Caregivers using the period pack contents	
		Expressed interest or intention to use	Participants reporting that they positively benefited from the campaign	In-depth interview, key informant interview
		Perceived demand		
			Participants and facilitators requesting involvement in future Veivanua campaigns	
**Implementation**	The extent, likelihood, and manner in which an intervention can be fully implemented as planned and proposed	Degree of execution	All training sessions were delivered as planned (e.g., following the facilitation guide, sessions were delivered in groups, the correct number of facilitators delivered the session, all necessary materials were available and distributed, all planned monitoring visits were completed)	Process monitoring tools, key informant interview
		Success or failure of execution		
		Factors affecting implementation ease of difficulty		
		Efficiency, speed, or quality of implementation		
		Efficiency, speed, or quality of implementation	Training sessions delivered within two hours	
**Practicality**	The extent to which an intervention can be delivered when resources, time, commitment, or a combination of these are constrained in some way	Positive / negative effects on target population	Perceptions of improvements across target behaviours	In-depth interview
		Ability of participants to carry out intervention activities		
		Amount, type or resources needed to implement	World Vision and facilitators do not consider the delivery costs to be prohibitive for humanitarian responses	Key informant interview
**Adaptation**	Changing the campaign contents or procedures to be appropriate in a new situation. It is important to describe the actual modifications that are made to accommodate the context and requirements of a different format, media, or population	Degree to which similar outcomes are obtained in new format	Improvements across target behaviours	In-depth interview
		Process outcomes comparison between intervention use in two populations	Veivanua campaign is acceptable to target groups, facilitators, and World Vision	In-depth interview, photovoice, observation, field notes, key informant interview, process monitoring tools

### Study site

SANMA Province, in northern Vanuatu, was the site of this study. The entire population has likely been affected by humanitarian crises. For instance, 8385 evacuees from the Ambae Volcano eruption in 2017 and 2018 lived in the province, and Tropical Cyclone Harold made landfall on Espiritu Santo (the largest island in SANMA) in 2020.

### Study population and sampling

Participants were identified through the formative research study sample [15]. Thirty young people were selected who had completed the campaign, were aged 15–31, menstruate and experience ‘a lot of difficulty’ or more across the ‘cognition’ functional domain in the Washington Group Short Set of questions [31]. All young people lived with their families. Thirty-five caregivers of young people were selected. All caregivers were relatives of the young person; 31 were women (22 mothers, nine aunts and sisters), and four were men (fathers). Key informants included two facilitators who delivered the intervention, two World Vision staff, and one disability service provider involved in the adaptation.

### Data collection methods

Facilitators administered process monitoring tools throughout the campaign delivery. These tools recorded the number of participants who had completed the training sessions, if these were delivered as intended (e.g., by following the facilitation guide, with the correct number of facilitators and if the required resources were available and distributed), to assess if participants were using the campaign resources to support the adoption of the target behaviours and, if not, how they could be supported to do so.

Recruitment for data collection started on 5 July 2022 and ended on 24 Augusts 2022. The research team applied several qualitative tools to enable methods triangulation: photovoice, in-depth interview, ranking and observation. S1–S3 File contain the topic guides for these methods.

Evidence is based on participants’ perceptions and self-reported data generated through several methods. Through in-depth interviews with nine young people and 35 caregivers, researchers explored changes in behaviours, responses to the training, use of campaign resources and their perceived appropriateness during an emergency. Caregivers were also asked to rank campaign resources according to which: a) the caregiver used most to least, b) the young person used most, and c) the caregiver is most to least likely to take when evacuating their home during an emergency. Additionally, researchers observed young people’s reactions when passed one campaign resource at a time. Responses, such as recognition, understanding of content or purpose, pleasure or dismissal, were recorded in field notes.

Photovoice is a visual research methodology applied in different settings to explore menstrual health, including during the formative research conducted before the campaign was adapted [13, 15, 17]. Participants own their photos and identify how they want to be credited when used. Two young people completed photovoice in this study and took photos about their experiences participating in the Veivanua campaign. Caregivers were involved in supporting the young person if required, but they were encouraged not to influence the young person’s choice of photo. Young people were interviewed about the photos to explore the meaning behind the photos, their feelings, thoughts and ideas when capturing the images, and to rank the images according to the most to least important topic. Caregiver’s opinions were sought to more fully understand the young person’s motives and behaviours.

Key informant interviews were conducted by LSHTM and World Vision staff who were not part of the facilitation team. Topics explored experiences of adapting the campaign for the new setting, training and support provided before and during the intervention delivery, running the training sessions, perceived appropriateness of the campaign and its resources in an emergency and their ability to integrate the campaign into existing humanitarian responses.

### Data analyses

Data were analysed iteratively. Those interviewing participants met at the end of each day to review their field notes, including observation data, reach a consensus about young people’s reactions to the campaign resources and minimise researcher bias. The broader research team met weekly to discuss emerging themes to ensure relevant topics were explored in future interactions.

Field notes were typed up, and voice recordings of interviews were translated and transcribed into English. Bislama-speaking research team members checked transcripts for accuracy; any errors were corrected before finalisation. All identifiers were removed from transcripts and field notes to ensure participant anonymity.

Researcher-led analyses of photovoice data were conducted to understand the campaign’s acceptability, demand and adaptation [32]. This involved thematically analysing content from the interview, exploring what the participant chose to capture in the photos and the importance they placed on them (ranking). The photo captions in this article were generated from words young people used to describe the photos during the interview.

Drawing on the following process set out by O’Connor and Joffe [33], all data were analysed thematically by 1) familiarisation with data, 2) conducting first-level coding against the ‘areas of focus’ in Bowen et al.’s feasibility study framework [29] applied in this study, 3) identifying additional codes, 4) developing a codebook for data organisation, 5) double-coding a small number of data to validate and improve the codebook content, 6) coding transcripts, 7) reviewing connections between codes and analyses across the whole research team. Data were organised and analysed in Nvivo 12.

### Informed consent process and ethical approval

**Fig 1:**
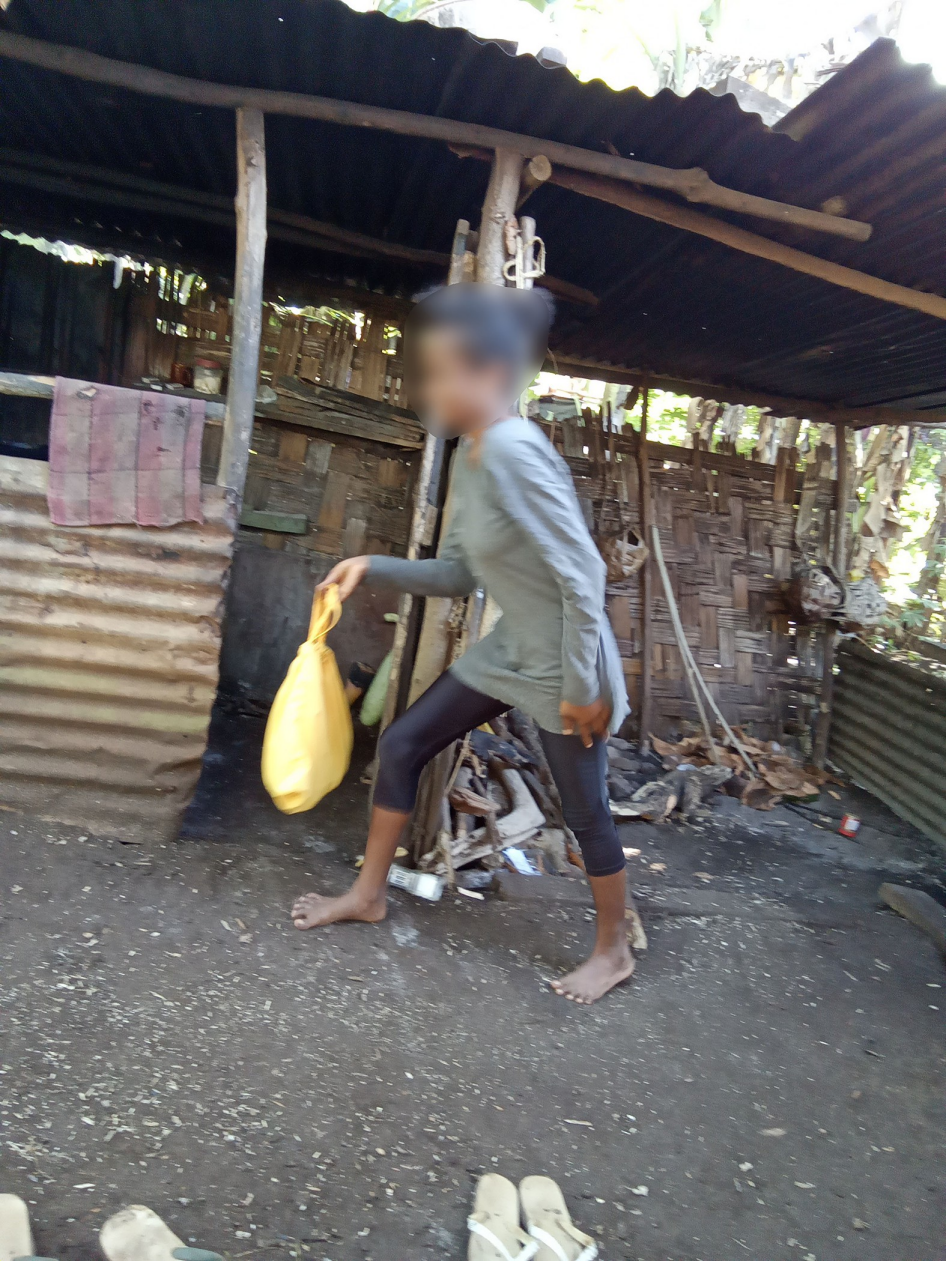
I’m going to a house to hide in case a cyclone comes”. **“** Josephine.

**Fig 2:**
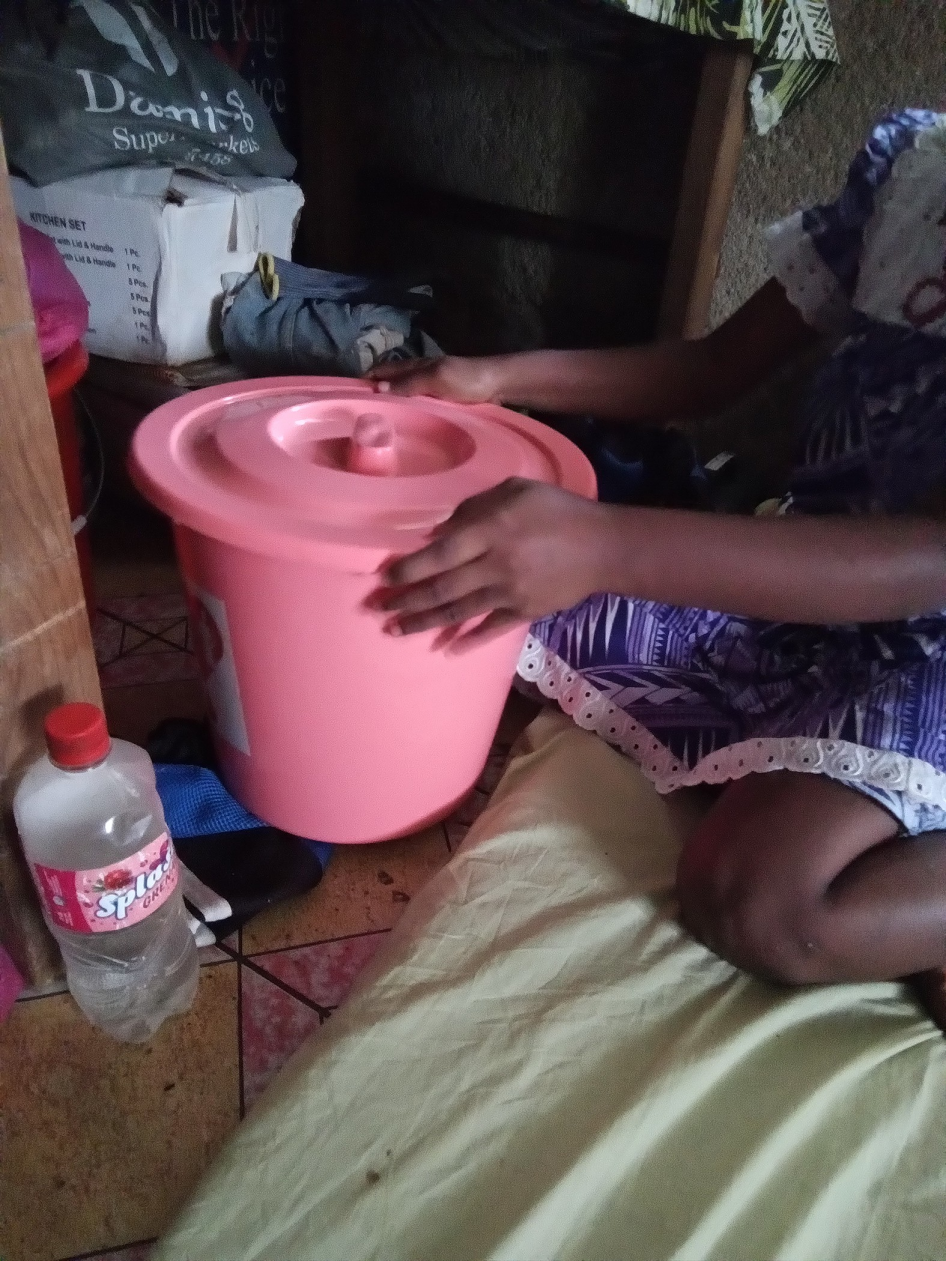
I’m shutting them in, so it stays in the bucket”. **“** Failine.

**Fig 3:**
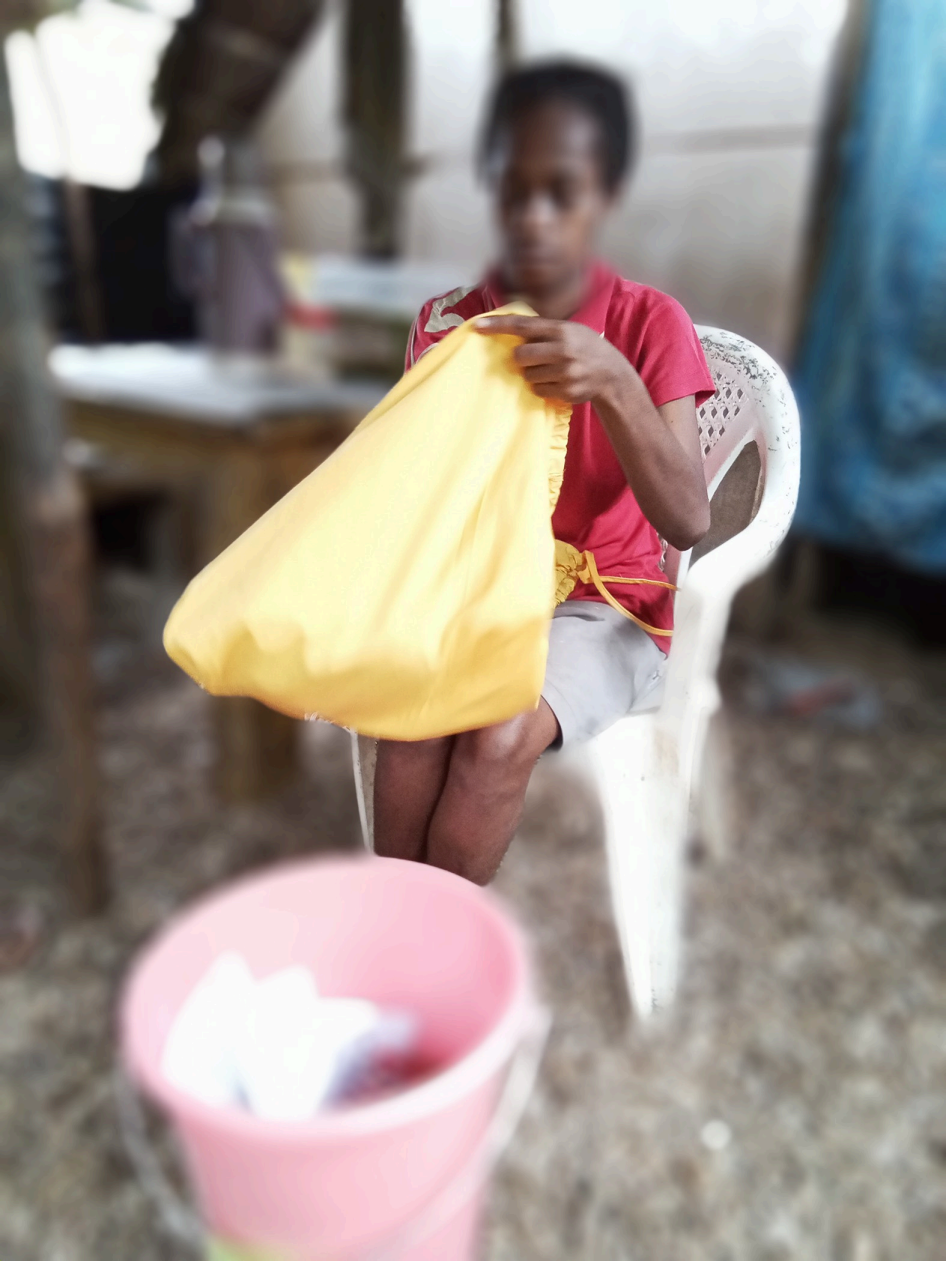
“Putting the calicos inside the bag. I like it”. Josephine. (‘Calicos’ refers to menstrual materials).

**Fig 4:**
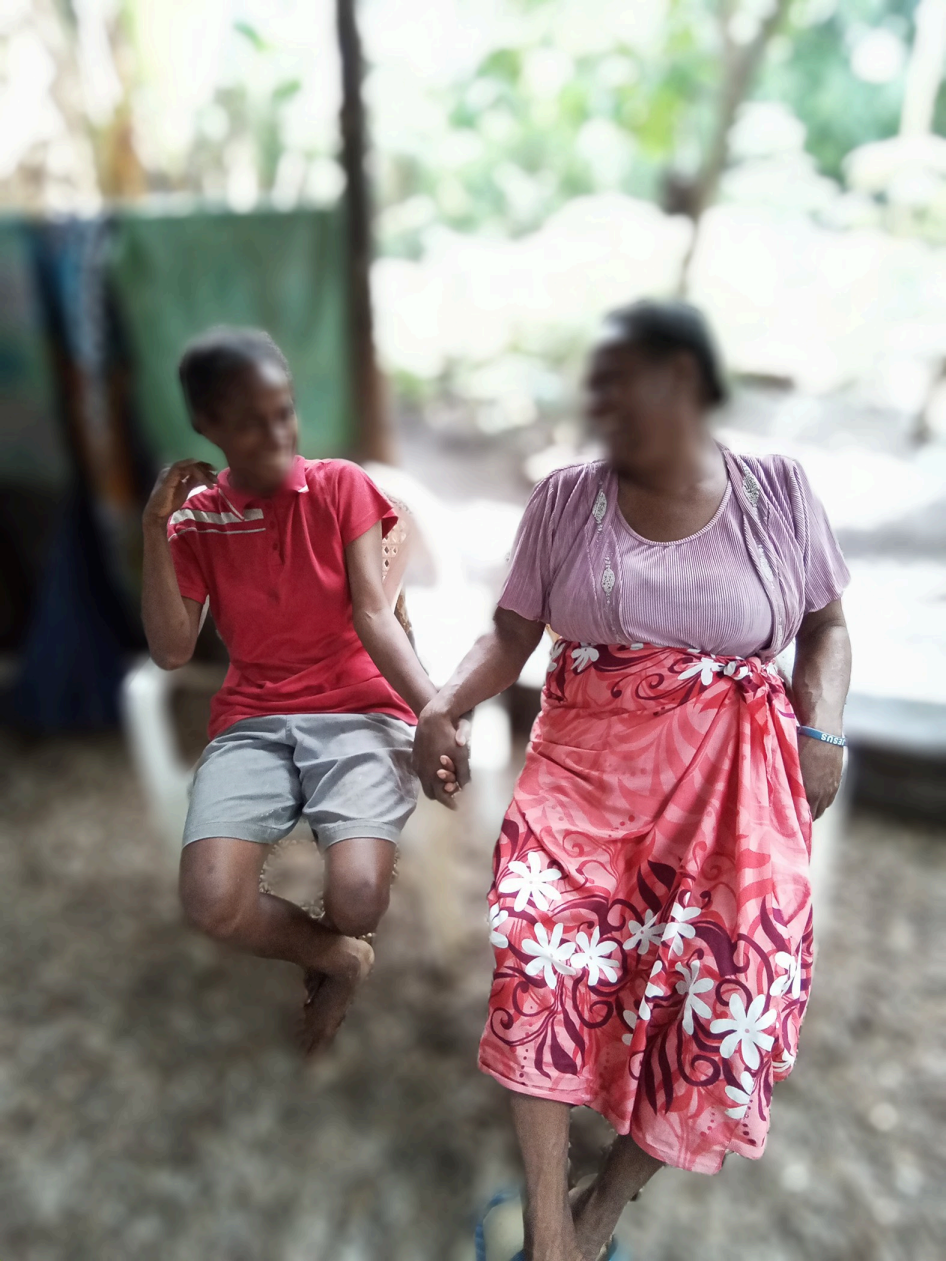
“I like to spend time with my Mama”. Josephine.

Informed consent was sought from all participants before enrolment. The researchers read the information and consent sheets in Bislama to participants. All caregivers and key informants provided written consent (thumbprint if illiterate). A simplified information sheet was read to young people, and caregivers supported their understanding. Caregivers provided consent for young people, who also gave their assent. The photovoice informed consent process was twofold: at the start of the process and again after the participant had taken the photos. Researchers explained that the participants own the pictures, asked if they would like to be credited with their real name or a pseudonym, and how they would like their photos used. The individuals pictured in Figs 1–4 have provided written informed consent (as outlined in PLOS consent form) to publish their image alongside the manuscript.

This study was approved by the Ethics Board of the London School of Hygiene & Tropical Medicine (code 26761). Vanuatu does not have an Ethics Committee, so endorsement for the study was sought and granted in writing from the Director General in Vanuatu’s Ministry of Justice and Community Services.

## Results

To ensure anonymity, pseudonyms are used with quotes instead of participants’ real names. One participant, Failine, who completed photovoice, wanted to use her real name.

### Acceptability

#### Satisfaction

All caregivers expressed how they valued being part of the Veivanua campaign; many said it helped them provide a higher quality of care for the young person.

“We’ve never had someone come to teach us these things–how to care for someone with a disability like this. When the campaign group came to teach us, they gave me some new ideas to work with.” (Vaiatea, female caregiver)

All male caregivers mentioned how the campaign enabled them to provide menstrual health care when the female caregiver is absent, noting how valuable that is.

“I am a male, you know, and when they were running this training, they were discussing women’s sickness [menstruation], and so I learnt things that I had never learnt, that I never knew, you know. And it has helped me. If sometimes her mother is not there, I can…” (Lono, male caregiver)

Most caregivers were satisfied with the length of the training sessions. For instance, one caregiver said, "the timing was just right; one hour was enough time to run a workshop with someone with a disability” (Joena, female caregiver). Yet a few would have liked the sessions to last longer so they could ask more questions. Interestingly, those who desired longer sessions reported that the delivery modes and information provided were clear and accessible, and the young person repeated training activities on their own small Veivanua doll.

“The instructions they provided were clear [….]. I was watching her, and I saw her try out everything that they taught her on the doll.” (Mary, female caregiver).

Caregivers noted that the role play enabled them to discuss menstruation more openly. Many found the technique enjoyable as it created a sense of fun when discussing a sensitive topic.

“When it was time for [the facilitators] to come, I was really afraid because I’m not a mother, then when they came and discussed, and we laughed, and we made the role play, it was good to laugh.” (Fiva, female caregiver, sister).

Many caregivers reported that the young person they support was pleased with the campaign resources, citing their engagement with the materials, especially the visual story and the doll.

“At dinner, morning, night, whenever… you can find her looking through the book. And they gave her a small doll, and she’s always playing with it. She washes the thing you put in the doll’s panties, hangs it to dry and then puts it back on in the afternoon. She’s always changing it.” (Christelle, female caregiver)

#### Intent to continue use and perceived appropriateness

Some caregivers said they used to consider menstrual health a minor issue. Yet, since completing the campaign, they recognised how supporting the young person to manage menstruation more independently, including during emergencies, benefits the whole family.

“It seems small, but taking these things with us [in an emergency] helps us so much, so much. The feedback helps us as mum and dad, but the family too”. (Aleki, male caregiver)

Many caregivers reported feeling more prepared for a future emergency: “The teachings are clear, and now we know how to handle a disaster, how to handle our daughter” (Lono, male caregiver). Many caregivers highlighted the importance of evacuating with period packs during emergencies.

“Sometimes when it is disaster time, everyone goes and [forgets about] her, and then when we get to the evacuation centre, and she sees her period, there is no material for her to use or to give her privacy, and people will say, ‘what is wrong with her?’ and we feel embarrassed. But [the training] reminds us carers to always be ready for disasters, to have everything to move with.” (Raiha, female caregiver)

Caregivers remarked that young people understood the training content and could explain what to do when the next emergency comes. One explained:

“They [facilitators] asked her what we would do if a hurricane were coming. Mahana said we would continue to listen to the radio and prepare by getting our bag of period products ready and the house sorted before leaving for somewhere safer. She also mentioned that she would take the small yellow bag with her, and because it is waterproof, she can take it anywhere we go, that way if she needs to use the bathroom while she’s on her period, she can use the yellow bag to keep her used pads until she gets home.” (Onaona, female caregiver)

When researchers asked Mahana if she had learned anything during the Veivanua campaign, she replied, “Yes, I have learned about periods.” Josephine, who participated in photovoice, also demonstrated an understanding of the campaign content by taking a self-directed portrait of her taking her menstrual bag to her kitchen in case of an emergency (Fig 1).

#### Organisational appropriateness

All facilitators were satisfied with the training on delivering the campaign. One key informant remarked: “That training prepared me. [It] was straightforward, so it was more about how I could put myself in those shoes to do it–to know how to facilitate the campaign.”

Facilitators and World Vision staff thought that aspects of the campaign could be delivered during all phases of humanitarian emergencies, including preparedness, immediate response, and medium to long-term recovery phases. One key informant explained that they plan to store period packs (the menstrual bag containing menstrual materials, underwear and soap) ready to distribute during the next emergency. Several key informants explained that they intend to integrate the Veivanua campaign into their work streams.

“We’re looking to take it and scale it further across all of our sort of area programs, so all of the geographical locations that we work in [will include] people with disability, with intellectual disabilities.” (Key informant)

Several key informants spoke about procuring and storing the period packs for emergencies. Currently, only one organisation produces reusable menstrual materials in Vanuatu, and one key informant highlighted the challenge of meeting demand:

“If we were to have them pre-positioned, you needed to have a whole heap of those made in advance to any prior disaster or emergency response occurring.”

### Demand

#### Actual use of the campaign components and perceived demand

Numerous examples of young people using the period packs were reported through observation, photovoice, and interviews with young people and caregivers (Figs 2 and 3).

Many caregivers said that they repeated the training session content with the young person at home to reinforce learning.

“Well, I was talking about the thing that they gave. And I said to her, ‘When you use it, make sure you don’t throw it all about. Use it and put it aside well, wash it and then use it again’.” (Elenoa, female caregiver)

Findings related to the menstrual calendar were mixed. Some caregivers were excited and interested in the calendar, reporting that it helped them track the young person’s menstruation. Others found the calendar confusing or unclear. An unintended consequence of the menstrual calendar was that several caregivers used it to predict the young person’s fertility and restrict her movement to protect her against unwanted pregnancies due to sexual violence.

“They drew up the calendar and showed us how to count the days, so I had a go myself and found that it did work. After talking with Mahana about it, she understands it too. She comes up to me at the end of her periods and says that she needs to stay at home, and I agree with her and tell her she’s to stay home for a week before she can walk around in the community again.” (Onaona, female caregiver).

When asked to rank the resources according to those most impactful for the young person, caregivers overwhelmingly identified the small doll, followed by the visual story. The bin was ranked as the least impactful. Caregivers identified the menstrual bag with reusable menstrual materials as the most impactful items and that they would take when evacuating. Many explained that the menstrual bag was particularly useful during an evacuation because many items could be put inside it, including all the campaign resources. When researchers asked Inina (a female caregiver) which item she would most likely take in an emergency, she replied: “Everything they gave, I would fill it all up and move with it". Overall, caregivers identified the calendar and bin as the least impactful and said they would be least likely to take the calendar and small doll in an emergency.

### Implementation

#### Delivered as intended

Originally, the training sessions were going to be delivered in groups. However, some participants could not attend the group sessions or felt uncomfortable speaking in front of a group, so the training sessions were delivered to people in their homes instead. Many facilitators spoke about the value of this approach and why it should be continued in the future.

“[Participants] don’t feel confident to ask something because they are shy and think they’ll ask a question that isn’t right. If we implement another campaign, I think it’s more appropriate to go from house to house.” (Key informant)

Conducting the training in homes meant that the content could be delivered according to the young person’s attention span and around the caregiver’s other commitments, including work.

COVID-19 disrupted implementation. Consequently, campaign delivery took longer than expected, meaning 10 participants were interviewed for the feasibility study within two weeks of completing the campaign. All household monitoring visits were conducted after the first training, but few were completed after the second session because of COVID-19.

### Practicality

#### Key behaviour. Young person: Use a menstrual material; Caregivers: Provide enough menstrual materials

Many caregivers reported that young people managed their menstruation more independently following the intervention. This included using a menstrual material, changing it regularly and washing hands with soap and water.

“The last month was the first time; we slept, and she went to the…toilet, and she saw (her period) and came and took her towel and went to bathe. […] I saw a big change in her because when we went, it was the first time ever…it’s helping her now.” (Pania, female caregiver)

Caregivers reported putting clean menstrual materials in the menstrual storage bag. This enabled young people to be more independent because they knew where to get clean menstrual materials.

“She used it all alone. Because it is there, she takes.” (Raiha, female caregiver)

Caregivers of young people who could not change their menstrual materials independently found storing clean menstrual materials in the menstrual storage bag helpful.

“When she was sick [menstruating], I would be looking all over for her things. But now it is here, it is safe.” (Kaamia, female caregiver)

#### Key behaviour. Young person: Do not show used menstrual materials in public

Findings reveal that more young people understand how to dispose of and reuse menstrual materials effectively, including putting them in the menstrual bin and washing and drying them in sunlight.

“She’d leave them [menstrual materials] all over the place […] It’s much better now. She uses them and then washes and folds them, and puts them back in the bag.” (Mary, female caregiver)

However, in the formative research, nearly half of the caregivers interviewed said they kept the young person at home during menstruation because they were concerned that they might leak menstrual blood or take their menstrual material off in front of others. After the campaign, no caregivers remarked that they would be more willing to allow the young person to leave home when menstruating than before the campaign delivery.

#### Key behaviour. Young person: Use pain relief; Caregiver: Provide pain relief; Show love

Limited results were found regarding the increased use of pain relief options, but some caregivers reported providing a greater variety of pain relief options. Caregivers reported an increased understanding of the cause of menstrual discomfort and how to respond.

“I see her just lie down, and I say to myself, ‘Maybe she has too much blood flow…’ But after the training, I realised that sometimes her belly is sore, or she has a headache, and you have to make some warm water and sit with her.” (Hali, female caregiver)

Several caregivers reported greater motivation to show love and support the young person when menstruating.

“I used to berate her: ‘Why are you doing this?’ I wasn’t taking care of her and looking after her. But through the training, I realised that she needed me. I look after her, and I teach her to look after herself and clean herself […]. Through the training that you have given me, I have changed her, and now people see her at another level.” (Lea, female caregiver)

Fig 4 captures the love and connection between her and her caregiver.

#### Amount and type of resources needed to implement

Key informants spoke of the cost of the period packs. Staff producing the campaign materials understood that high campaign costs would inhibit scale-up, so they “tried to keep the packs at no more than 10,000 Vatu” (approximately 85 USD). Many key informants highlighted that Vanuatu depends on imports, making development interventions and humanitarian responses especially costly.

Table 2 presents the cost of a Veivanua campaign period pack ($57 USD), which could be prepositioned for a humanitarian emergency.

**Table 2:** Cost of a Veivanua campaign period pack.

Item	Unit cost (UDS)
Menstrual storage bag	$ 13
Menstrual materials	$ 26
Small drawstring bag	$ 11
Visual story	$ 7
**Grand total**	**$ 57**

Table 3 compares the production costs of the Bishesta and Veivanua campaigns, excluding the one-off costs, such as salaries for artists to produce campaign visuals. Salaries and delivery costs are excluded because the unit costs and sample size differed between countries. Table 3 shows that the overall costs were slightly higher in Nepal, even though several resources and training materials were excluded from the Veivanua campaign. This is because the production costs were considerably higher in Vanuatu than in Nepal, as demonstrated by the 93% increase in the small doll unit cost in Vanuatu.

**Table 3:** Comparison of Bishesta and Veivanua campaign production costs.

Category	Item	Bishesta campaign unit cost (USD)	Veivanua campaign unit cost (USD)	Cost increase (%)
**Campaign resources**	Tailor made menstrual pads	6.50	26.00	300
	Menstrual shoulder / drawstring bag	5.69	11.00	93
	Large menstrual storage bag	11.30	13.00	15
	Pain scale bracelet	1.32	N/A	N/A
	Bin	4.59	8.77	91
	Menstrual calendar	2.10	0.09	-96
	Small doll	18.20	35.09	93
	Visual story 1	3.60	6.89	91
	Visual story 2, Mirror, dangles, key rings, badges	11.50	N//A	N//A
**Training materials**	Large doll	59.02	61.40	4
	Masks for the game of life x8	3.80	7.04	120
	Key behaviour cards	4.00	8.77	119
	Campaign banner, bunting, printed visual instructions, bag for facilitator	53.30	N//A	N//A
**Grand total**	** **	**184.92**	**178.05**	
**Cost per young person (n = 30)**		**6.14**	**5.94**	

#### Adaptation

Several modifications were made to adapt the Bishesta campaign for Vanuatu’s humanitarian context. Firstly, the age of young people with intellectual disabilities who participated in the campaign was extended from 15–24 to 15–31 years. The purpose was to increase the sample size from ten in Nepal to 30 young people in Vanuatu.

Analyses of formative research data demonstrated that the Bishesta campaign target groups and behaviours were relevant in the new context. However, to address the concern that the target behaviour, ‘do not show blood in public’, may have unintentionally reinforced shame around leaking menstrual blood on clothes in Nepal, the wording was changed to, ‘do not show used menstrual materials in public’. This new behaviour more explicitly relates to changing menstrual materials in private.

The campaign visuals were amended to reflect the socio-cultural context and a humanitarian setting. Bishesta was a young woman with Down syndrome. Few participants in the formative research in Vanuatu had Down syndrome, so nor did Veivanua. The story depicted the family preparing for a cyclone with Votahenavanua and Veivanua’s father helping her pack her menstrual materials and evacuating with her period pack. In the Veivanua visual story, the father plays a central role in supporting menstrual health. This was not the case in the Bishesta campaign, as the formative research found that only women provide menstrual care in that setting.

Several changes were made to the Bishesta campaign resources to reduce costs. One visual story, instead of two, was produced because one would be easier to distribute in an emergency. This also reduced printing costs. A simpler small drawstring bag replaced the shoulder bag; the campaign mirror and key rings handed out to participants in Nepal were not manufactured, and the Bishesta campaign banner, which depicted the target behaviours and was placed at the entrance to the group training venue, were not produced. In Nepal, the pain bangle was not understood by participants, so this was excluded from the Veivanua campaign. Finally, the menstrual calendar was considerably simplified in Vanuatu to support greater understanding. Regarding delivery, in Nepal, the facilitation team that delivered the campaign included caregivers of young people with disabilities. In Vanuatu, facilitators included a woman with disabilities and a caregiver.

The Bishesta campaign was delivered with more fidelity than the Veivanua campaign, but both were acceptable to the target groups, facilitators, WaterAid, World Vision and disability service providers. Most of the period pack contents were used by participants, and improvements were recorded across all target groups. However, improvements in the target behaviours of the Nepali participants were more marked. The delivery of both campaigns led to increased and more open communication about menstruation between young people and caregivers. Caregivers in both settings reported a greater understanding of menstruation and how to provide dignified menstrual care, contributing to increased confidence.

Unintended consequences of the campaign were observed in both settings. In Nepal, caregivers noted increased confidence in young people to manage their menstruation, leading parents to view them as more “grown-up”. In Vanuatu, many caregivers used the doll to teach young people about their bodies and social skills.

“Sometimes, I would say. ‘You want to shake hands with Veivanua?’ she would not say anything, and then I would move her hand towards the dolly to touch her, and I would say, ‘That’s how we shake hands.’ [….] I’ll point out the mouth, the eyes, and I’ll say those are hers, and those are yours. She has seen it so often that now it is opening up her mind.” (Hana, female caregiver)

## Discussion

This study shows that the Veivanua campaign is feasible and acceptable. This is evidenced by caregivers stating they can better support young people to manage menstruation as independently as possible and are more prepared for the next emergency, key informants’ intent to deliver the campaign in their existing humanitarian responses, and the improvements recorded across all target behaviours. The intervention was not delivered as intended, but the revised mechanisms suited the context. The Veivanua campaign cost was higher than the Bishesta campaign, but this is because procurement is more expensive in Vanuatu. Several changes were made to adapt the Bishesta campaign for the socio-cultural and humanitarian settings, and similar outcomes were observed in both contexts.

### Acceptability

Assisting caregivers of persons with intellectual disabilities was identified as a major gap in supporting menstrual health for this population in numerous development and humanitarian settings [12, 14, 15, 18]. This study begins to fill that gap, and caregivers noted that they thought the Veivanua equipped them to provide a higher standard of care than previously. This was also found in Nepal. Both feasibility studies cited the target behaviour, ‘show love and emotional support’ and informing caregivers about how to support young people’s menstrual health as catalysts for change.

As in Nepal, the delivery mechanisms and information were accessible to everyone [27]. In both campaigns, caregivers remarked how useful role-play was to put people at ease and increase communication about menstruation. In Vanuatu, more young people repeated training activities unprompted at home with their small dolls, whereas in Nepal, caregivers initiated such exercises. The difference may be that most young people in Nepal lived in a residential care setting, so they relied more on staff to direct their activities.

Results from the formative research conducted before delivering the Veivanua campaign revealed that caregivers’ desire to maintain their young person’s safety and privacy limited their evacuation choices [15]. In this study, caregivers remarked that they recognised the importance of evacuating with the young person’s period packs to support her privacy and dignity; and that the knowledge gained and campaign resources enhance emergency preparedness. The Veivanua campaign also supported young people in understanding what to do during an emergency, and many practised evacuating home with their period packs. Routines can create order and are important for stability and continuity [34]. During cyclones, when outcomes are unpredictable, equipping young people with these new routines may create a basic sense of order during an evacuation. This hypothesis needs testing.

During the formative research, key informants called for a menstrual health intervention for people with intellectual disabilities that could be implemented before, during and after an emergency [15]. Following the delivery of the Veivanua campaign, facilitators, World Vision staff, and key informants reported that the Veivanua campaign was appropriate for the humanitarian context. World Vision Vanuatu plans to train the Vanuatu Society for People with Disabilities (the only disability service provider operating nationally in Vanuatu) staff to integrate it into their self-care and disaster response programmes in 2023. This provides further evidence of campaign acceptability. Furthermore, World Vision Vanuatu has secured additional funding to scale up the campaign in the country. Specific guidance about how the campaign could be delivered in the different phases of humanitarian responses, from preparedness to long-term recovery, is published elsewhere [35].

### Demand

The small doll and visual story were identified as the most impactful for triggering the young person’s behaviour change. This was also recorded in the Bishesta campaign’s feasibility study [27]. However, caregivers said they would be least likely to evacuate with the small doll during an emergency. This could be because the doll could get lost or not considered an essential item. Interestingly, caregivers identified the large menstrual storage bag containing reusable menstrual materials as the item they would take during an evacuation, partly because they could store other items in it, including all other campaign resources. Therefore, the prepositioned period packs should be the large bag containing the reusable menstrual materials, the visual story and the small drawstring bag. It would not include the menstrual bin or calendar because these were not ranked highly for impact.

As stated in the Sphere Handbook, humanitarian needs do not align with specific sectors: WASH, including menstrual health, food security and nutrition, shelter and settlement and health [7]. Distributing the Veivanua period packs does not negate the need for other items crucial for survival, such as food and non-food items (e.g., drinking water, water containers, sanitation, soap, temporary shelter, blankets, cooking utensils). Coordination and collaboration across sectors and efforts are vital to effective humanitarian responses.

The Bishesta campaign menstrual calendar was difficult to understand, so it was considerably simplified in the Veivanua campaign. Yet, participants of the Veivanua campaign also found it confusing. Following the feasibility study, the research team reflected that using calendars outside professional settings is not widespread in Vanuatu, especially in rural areas. The purpose of the menstrual calendar was to trigger the caregiver’s behaviours. For instance, caregivers would use the calendar to track the young person’s menstrual cycle. When menstruation was approaching, the caregiver would fill the large menstrual bag with materials and remind the young person of their target behaviours shortly before they menstruate. The calendar was not intended to track fertility and restrict the young person’s movements.

Globally, people with disabilities are more vulnerable to abuse than people without disabilities; women and girls with disabilities and people with intellectual disabilities face the greatest risk [36–40]. The formative research highlighted that some study participants with intellectual disabilities had been sexually abused when they were unaccompanied outside the home [15]. This led their caregivers to keep them at home or ensure they were never alone in public, especially when menstruating. Within this context, it is less surprising that caregivers used the menstrual calendar to track the young person’s fertility. Still, it does highlight the importance of delivering programmes to combat disability discrimination and promoting the SRH of persons with disabilities alongside the Veivanua campaign and ensuring they are accessible and inclusive to people with disabilities. Generally, the purpose of SRH interventions is to prevent gender-based violence and aggressive behaviour and reduce unwanted or high-risk pregnancies and sexually transmitted infections [41], all of which are relevant for the ni-Vanuatu setting as well as all other countries in the world.

Furthermore, data generated through the formative research in Nepal and Vanuatu show how multiple power systems operate within and across diverse social settings. These are revealed through common experiences of participants, including participation restrictions during menstruation and physical, verbal and sexual violence [15, 17, 25]. By applying an ‘intersectionality’ lens to analyses, it becomes apparent how various factors such as disability, impairment experienced, gender, age, and sociocultural norms are interlinking forms of oppression, discrimination and disadvantage [42–44]. As broader social and structural factors shape and drive behaviours, intersectionality must be considered within menstrual health.

### Implementation

The Veivanua campaign was not delivered as intended for several reasons. Firstly, the delivery was interrupted by cyclones and COVID-19. Secondly, many participants felt uncomfortable in the group setting, and caregivers needed to seek work to aid post-disaster recovery. This proves the pilot study’s value because the campaign was designed for the humanitarian context and implemented during emergencies. As highlighted in the formative research, the ability to work after an emergency is vital for recovery, so delivering the training around caregivers’ employment opportunities in their homes supported this [15]. In Nepal, group training worked, but several participants lived in a residential home. Many knew each other, which may have facilitated a greater willingness to engage in group discussions. Participants also lived closer to each other than in Vanuatu (where some participants were reached by boat), and all could get public transport to the campaign training venue.

### Practicality

Improvements were recorded across all target behaviours. However, in Vanuatu, even though more young people used and reused or disposed of menstrual materials effectively, this did not positively impact the caregiver’s willingness to allow the young person out of the house when menstruating. In Nepal, harmful socio-cultural norms related to menstruation are widespread and internalised [45]. Women and girls with disabilities and caregivers worried they would be cursed if these norms were not followed [17]. Some people with intellectual disabilities who did not wear a menstrual material and therefore leaked menstrual blood onto their clothes were abused for doing so. Consequently, caregivers kept the young person at home when menstruating. After the Bishesta campaign was delivered, caregivers were likelier to let young people out of the home when menstruating because more were wearing menstrual materials and changing them regularly [27]. Though menstrual taboos exist in Vanuatu [13], the driver for keeping young people at home when menstruating was to protect them from gender-based violence [15]. This concern was not addressed through the Veivanua campaign and may explain the reason for these different results.

The production of the Veivanua campaign resources and training materials was more expensive than the Bishesta campaign, meaning that although several components were excluded in Vanuautu, the overall cost per young person was similar to Nepal. In Cyclone Harold, one of World Vision’s family hygiene kits, containing items such as soap, laundry soap, toothpaste and toothbrush, sanitary napkins, women’s underwear, washable baby diapers and pins, cost approximately $65 (including freight). Therefore the cost of one period pack ($57 USD) is comparable and would complement the distribution of standard hygiene kits. Like most other countries in the Pacific, Vanuatu has one of the lowest purchasing index scores globally [46]. This, along with its geographic remoteness, limited to no internal manufacturing capabilities and dependence on imports of basic goods and supplies for its population living across 83 islands, makes Vanuatu an expensive setting to procure items for poverty reduction programmes [46]. These dynamics intensified during COVID-19 as costs increased further because of disruptions to the global supply chain.

### Adaptation

Several modifications were made to adapt the campaign to Vanuatu’s humanitarian context. Like the Bishesta campaign, the Veivanua campaign was acceptable for target groups and key informants, and improvements were observed across all target behaviours. Unexpected positive outcomes were observed in both settings, demonstrating that these campaigns could be an entry point for wider self-care support for women and girls with intellectual disabilities. It is encouraging to see that the outcomes across the two campaigns are similar, even though several campaign resources were excluded from the Veivanua campaign. This demonstrates that a slimmed-down and cheaper campaign version could be adapted and delivered in a humanitarian setting within a country that does not rely so heavily on imports. This hypothesis needs testing.

### Strengths and limitations

A key strength of this study is that it followed a systematic process to adapt the Bishesta campaign for the new setting, meaning it is evidence-based and contextually relevant. Most young people and their caregivers participated in the formative research, the campaign and the feasibility study. Therefore, participants’ requirements shaped the adaptation of the campaign, which could have increased its acceptability for the target groups. A major strength was that the Veivanau campaign facilitation team included a woman with disabilities, which aligns with the disability principle, ‘nothing about us, without us’ [47]. Approaches that ensure women with disabilities participate in interventions that are intended to benefit this population increase the likelihood that interventions are delivered in a way that is inclusive and acceptable.

Regarding limitations, the authors used the Washington Group Short Set of Questions to gauge the cognitive abilities of people with intellectual disabilities. These questions are validated and widely used in population-based disability surveys, yet they have limitations [48–50]. For instance, by only answering ‘a lot of difficulty’ or ‘cannot do at all’, the complexity of intellectual disability is not captured. Consequently, some authors call for a combination of self-report and clinical assessments that rely on clinicians to diagnose impairment, but this was outside the resources of this study [51–53]. Additionally, this was the first time caregivers had been supported to provide menstrual health for young people. Consequently, there is a potential that caregivers’ responses to questions in this study were overly positive due to a lack of other comparable interventions. This risk of bias was managed by researchers asking open and probing questions to ensure participants could critique the campaign, close supervision and guidance provided by the LSHTM, and methods and data triangulation.

### Implications for further research

The following areas of further research are required.

- Feasibility studies effectively assess if an intervention merits further investment and efficacy testing [29, 54, 55]. This study has shown that the Veivanua campaign is feasible in Vanuautu’s humanitarian context, so now needs efficacy testing through an impact evaluation before scaling up.
- Global priority research questions for water, sanitation and hygiene (WASH) in humanitarian emergencies have recently been published [56]. Out of 128 research questions, adapting WASH programmes and services for people with disabilities and improving menstrual health in emergencies were identified as the second and fourth research questions, respectively (p13) [56]. As the Veivanua campaign is feasible in Vanuatu, it should be adapted again for another humanitarian context in a different country by applying this study’s research design. Evidence generated from this study and another setting would contribute to answering the two global research priorities.
- The Veivanua campaign is based on experiential learning theory (whereby people learn through experience) [57]: participants learn how to manage menstruation using the Veivanua doll. During the campaign, information is communicated in a variety of ways to ensure it is accessible. Therefore, the campaign could be adapted for people with visual and hearing impairments in Vanuatu’s humanitarian responses, following the systematic research process applied in this study.

## Conclusion

This study aimed to assess the feasibility and acceptability of the Veivanua campaign by investigating its acceptability, demand, implementation, practicality and adaptation. It found that the campaign was feasible for caregivers, facilitators, and practitioners in the humanitarian context in Vanuatu. Improvements were recorded across all target behaviours; participants indicated that they would evacuate with the period packs in the next emergency and that the campaign has made them feel better prepared. Male and female caregivers noted an increase in their ability to provide better-quality menstrual care and the young person’s ability to manage menstruation more independently.

This study adds to the limited literature about menstrual health interventions for women and girls with intellectual disabilities and their caregivers. Still, it is the first designed for the humanitarian context. Therefore, it begins to fill a significant gap, but much more must be done. The Veivanua campaign requires efficacy testing in Vanuatu and adaptation for emergencies in other countries to achieve the menstrual health of this previously excluded population.

## Supporting information

S1 File Photovoice topic guide for young people. (DOCX)

S2 File Topic guide for caregivers. (DOCX)

S3 File Topic guide for interviewing and observing young people. (DOCX)

S1 Text Inclusivity in global research. (DOCX)
