# Supplementary material for: Feasibility study of a menstrual health behaviour change intervention for women and girls with intellectual disabilities and their caregivers for Vanuatu’s humanitarian responses
Source: PLOS Glob Public Health. 2024 Jan 19;4(1):e0002244. doi: 10.1371/journal.pgph.0002244 (PMC10798467; doi:10.1371/journal.pgph.0002244)
Supplement: S1 File — (DOCX) [file pgph.0002244.s001.docx]

PhotoVoice guidance for young people

Title of the project: Feasibility study of a menstrual hygiene management programme for women and girls with intellectual disabilities and their caregivers in Vanuatu

# PhotoVoice Guidance

## First meeting

**Seek initial informed assent from the young person and consent from the caregiver. If granted, continue.**

**Understanding photography** - Many participants may never have seen a camera before or any (or few) photos, so it is important to explain the purpose of photography. Tell the young person that photos capture a moment as if it is real. Take a photo and show it to her. Give the young person the camera; show her how to take a photo. Let her take some photos. Talk to her about what she has taken [be positive and congratulate her on taking photos]. Teach the caregiver the basic features of the device including how to switch the camera off and on, how to take a photo and how to view the photo, how to change the battery.

**Run through this with the caregiver: Taking photos without showing a face to protect the young person’s identity** – people may wish to hide their own and other's identities, so you will need to explain how to do this. Remind the caregiver that we will blur anyone’s face in the photos, but say that these are other techniques:

- Taking photos with the light behind the subject so the figure will be in shadow.
- Focusing on something behind or in front of the subject. This means that the subject will be in soft focus
- Photographing a person's shadow
- Taking a photo of someone from behind (the back of their head, their head / body etc)
- Not taking photos of their own house

**Ensuring photos protect the participant’s dignity** – explain that if the young person takes photos of menstrual ‘accidents’ that these will not be used. This includes clothes, bedsheets or other materials with blood

**The photographic task**

Take five photos of things that make you happy. Then take five photos that represent your experiences of the Veivanua campaign. This may include the campaign resources and/or how you use them.

Lay the campaign resources out in front of the participant. Work through an example with the young person. Stand back whilst she takes the photos. Offer guidance, but don’t lead her to take specific photos.

**Self-Directed portraits** - It is likely that the young person will want to be in photos instead of just taking them. If that’s what she wants, she still must tell the researcher or caregiver how to take the photo, what is included in the photo, how much of their body should be in shot etc.

**Arrange a suitable time to return to the individual’s house to give them their photos and have a short discussion. Send the photos to the team leader for review, and arrange a time to discuss the photos.**

## Second meeting

This second meeting will take up to one hour. Run through the information sheet and seek their assent/consent. Don’t go through the consent checklist until the end of the interview. Show the participant the printed photos. Discuss each one, following the guiding questions below. Seek final assent/consent to use the photos by going through the consent checklist. It is impossible to do guiding questions for PhotoVoice as we don’t know what participants will take photos of, so be flexible and responsive to what they say.

**INSTRUCTIONS: INTERVIEWER TURN ON THE TAPE RECORDER AND SAY CLEARLY THE DATE, TIME, LOCATION, AND THE INTERVIEWER’S NAME**

- Go through one photo at a time. Ask all these questions for each photo

1. **Here are your photos. Which photo shall we talk about first?**
2. **What is this photo of?**
   - Why did you take this photo?
   - Why is this important to you?
3. **Move to the next photo and repeat the process.**
4. **Ask if [name] has any questions for you. Thank her. Leave the printed photos with her**
5. **Go through the secondary consent checklist with [name] and her caregiver**
