# Supplementary material for: Feasibility study of a menstrual health behaviour change intervention for women and girls with intellectual disabilities and their caregivers for Vanuatu’s humanitarian responses
Source: PLOS Glob Public Health. 2024 Jan 19;4(1):e0002244. doi: 10.1371/journal.pgph.0002244 (PMC10798467; doi:10.1371/journal.pgph.0002244)
Supplement: S2 File — (DOCX) [file pgph.0002244.s002.docx]

**Feasibility study of the Veivanua campaign:**

**Topic guide for caregivers**

**INSTRUCTIONS: INTERVIEWER TURN ON THE TAPE RECORDER AND SAY CLEARLY THE DATE, TIME, LOCATION, AND THE INTERVIEWER’S NAME**

**Rapport building questions**

- Introduce yourself and your role in the research
- Ask about the person’s family
- If they are a professional carer, ask about their role, how long they have worked there

Now I want to ask you about the Veivanua campaign. We are reviewing the campaign to understand what has and has not worked so that we can make it better. As you have been part of it, we are interested to hear what you think. Please be as open as possible. There are no right or wrong answers. What you say will really help us improve the programme.

**Relationship between the carer and the person they look after**

1. **What is your relationship to the person you look after?**
2. **Could you tell me a bit about [female’s name]?**

- How old is she?
- What does she enjoy doing?

1. **How long have you been caring for [female’s name]?**

**Behaviour change**

1. **Since completing the Veivanua campaign, have you noticed any changes in how [female’s name] manages her menstruation, and how she feels about it? Probe into:**

- Changes in use of a menstrual material [e.g. wearing one, changing it more regularly, disposal or re-use, handwashing, not taking it off in front of others]
- Changes in using pain relief options
- More comfortable or confident when menstruating

1. **Have you noticed any changes in the way you support (female’s name's) menstruation, or view it after being part of the campaign? Probe into**

- Changes in preparing for the young person’s next menstruation [putting menstrual materials where the young person can get them herself]
- Changes in providing pain relief options
- Greater communication about menstruation between the carer and young person
- Feeling more comfortable and confident / feeling less frustrated or angry
- More willing to take the young person out of the home when menstruating

**Campaign training sessions**

1. **Can you tell me about your involvement in the Veivanua campaign?**

- Did you attend group campaign sessions or did someone visit you in your home?
- Did [female’s name] go with you to all the sessions? (If not all, ask which the young person did attend)

1. **Can you explain what happened at the group campaign sessions [or the household visits]?**
2. **What were your expectations of the sessions? Please explain.**

- Were these met?
- What did you think of the content?
- Did you think it was helpful? Please explain.
- *If the participant did not attend the group campaign sessions, skip to question 10*

1. **How did you and [female’s name] get to the campaign sessions?**
2. **Who made the decision that you could attend the campaign sessions?**
3. **How did you and [female’s name] feel about the time spent at the campaign sessions / household visits [e.g. were the sessions too long, too short, or just right]?**
4. **Did you feel comfortable at the campaign sessions / during the household visits? Please explain**

- Probe into reasons for the answer given

1. **As you know, this campaign is designed to be delivered during or after emergencies. Were you given re-usable pads or period pants in the large menstrual storage bag?**

- What did you think of these materials?
- What did you think of the instructions on how to use them? Were they clear, or unclear?

1. **How many menstrual materials [re-usable pads or period pants] did you receive in the large menstrual storage bag? What do you think about this number?**

- Would the number provided mean you could wash and dry them quickly enough, even during heavy rains?

1. **Beyond what you have already told me, how does the Veivanua campaign compare to those other menstrual health interventions [and/or hygiene kits] you have received in past emergencies? Probe into:**

- Their levels of satisfaction with the contents of the large menstrual storage bag compared to other menstrual hygiene kits delivered during emergencies

1. **Having taken part in the campaign, is there anything you will do differently in relation to [name’s] menstruation during another emergency? Try not to lead the participant, but probe into:**

- If they will use the Veivanua campaign hygiene kit?
- If they will consider the young person’s menstruation more during emergencies?
- If they feel better prepared now?
- If/how the campaign will the caregiver more easily manage the young person’s menstruation in another emergency
- If/how the campaign will help the young person manage menstruation during another emergency

***Question for caregivers of young people who have used the period pants***

1. **What do you think of the period pants? Probe into:**

- Did the young person wear them?
- Was the young person more comfortable using them compared to other menstrual materials? If so, why and what makes the caregiver think that?
- Did the caregiver and / or young person face any challenges using them?
- What menstrual material they will use in the future and why?

**Resource assessment**

Now I’m going to ask you about the campaign resources. Please give as much detail as you can as this will help us improve the materials. I have them all here, so you can pick them up and look at them as you talk.

*Notes for researcher:*

- *Layout the resources in front of the carer*
- *It is important that the carer gives her own thoughts on the resources, and explains what the person s/he cares for thought of them. So for each of these questions, please ask the carer for her/his own thoughts and for the thoughts of the person with an intellectual disability.*
- *When the carer identifies the resource, verbally repeat the selection so it is captured on audio. Do this subtly so you don’t disrupt the flow of the conversation.*
- *If a number of resources have been used, ask these questions on all of them separately*

1. **Have you or [female’s name] used any of the resources?**

- Please show me the resources you have used

1. **Which resource did you use most? Please explain why**

- How did you use it?
- Why did you use it so often?

1. **Now, looking at the campaign resources, please can you put them in order of those you used most to least often? Left being the most used and right being the least used.**

- *Say the order into the voice recorder so we understand exactly how each caregiver has ordered the campaign resources from least to most used.*

1. **What did you like about it?**
2. **What did you not like about it?**
3. **How could it be improved?**
4. **Now, please order them according to those liked best to the least? Left being the most liked and right being the least liked.**

- *Say the order into the voice recorder so we understand exactly how each caregiver has ordered the campaign resources from least to most liked.*

1. **Which resource did [female’s name] use the most? Please explain why**

- How did [female’s name] use it?
- Why did [female’s name] use it so often?

1. **What did [female’s name] like about it?**
2. **What did [female’s name] not like about it?**
3. **Were there any parts of it that [female’s name] did not understand? Please explain**
4. **What did you and the young person think about the menstrual material/s [e.g. reusable pads, period pants] in the large menstrual storage bag?**

- What did they like and not like about the different menstrual materials? Why?

1. **Which, if any resources would you take with you during an emergency? Why?**

- Probe into which resources they would not take and why?

1. **Now can you order the resources according to those you would take with you during an evacuation? Left being the most likely to take and right being the least likely to take.**

- *Say the order into the voice recorder so we understand exactly how each caregiver has ordered the campaign resources.*

Thank you very much for your thoughts on the programme and for answering all my questions. Those are all my questions. Is there anything else you want to tell me? Is there anything you would like to ask me?
