# Supplementary material for: Feasibility study of a menstrual health behaviour change intervention for women and girls with intellectual disabilities and their caregivers for Vanuatu’s humanitarian responses
Source: PLOS Glob Public Health. 2024 Jan 19;4(1):e0002244. doi: 10.1371/journal.pgph.0002244 (PMC10798467; doi:10.1371/journal.pgph.0002244)
Supplement: S3 File — (DOCX) [file pgph.0002244.s003.docx]

**Feasibility study of the Veivanua campaign:**

**Guide for interviewing and observing young people**

**IN-DEPTH INTERVIEW QUESTION GUIDE**

**Objective**: to explore what the young person thought about being part of the Veivanua campaign. In particular, to investigate her thoughts and feelings about each campaign resource.

**Inclusion criteria**: young person who participated in the Veivanua campaign.

**Materials needed:** information and assent sheet, voice recorder, spare batteries, notebook and pen, all the campaign resources (large menstrual storage bag, small menstrual bag, I Manage visual story, large Veivanua doll, menstrual bin), Vetahenavanua campaign masks (happy and sad) and refreshments.

Activities

EMOTIONS AND DRAMA

**Participant: a person who can recognise visual cues and emotions**

**Materials needed: Vetahenavanua happy and sad campaign masks, large Veivanua doll, ‘I Manage’ visual story, large menstrual storage bag, small menstrual bag, the menstrual bin**

**Process:**

Show the young person the happy and sad masks

Encourage her to look at each expression

When she picks one up, ask her how she thinks Vetahenavanua is feeling. Have fun. Enact out the expressions / emotions with her.

Line the masks in front of the young person

Give her the large Veivanua doll

Observe her reaction to it. If she smiles when she sees the doll or picks it up and hugs it, you know that she may like the doll

**Observer**: record her reaction

Ask her to point to the mask that represents her feelings about the Veivanua doll

If she is unsure, you can go through the masks together. Ask her if the doll makes her happy (show the happy mask)? Sad (show the sad mask)? If she says yes to all the emotions, you know that she is just agreeing with what you say instead of understanding the emojis

Ask her again which mask represents her feelings towards the large Veivanua doll

**Observer**: record the young person’s reaction and which mask she identifies

Ask her questions related to the chosen mask. For instance, what do you like about it; what makes you sad?

**Observer**: record her responses

Repeat the exercise for the following campaign components in this order (work through as many as you can, but don’t worry if you don’t get through them all):

‘I manage’ visual story

The large menstrual storage bag

The small menstrual shoulder bag

Menstrual bin

Ask if she wants to tell you anything else?

Close the session

**After the interview, discuss your notes and how you thought the young person responded to each resource with your co-interviewer. Did you both interpret the young person’s reaction to each resource similarly? If not, discuss this. Finalise your notes and type them up.**

**ALTERNATIVE SESSION**

Some young people might get too distracted by the face masks. If they do, do not use them. Instead:

Pass each campaign resource to the young person one at a time.

Observe her reaction to the campaign resource and write this down.

If she is responsive, ask her questions about each resource after you have passed it to her, such as what does she like about it and why?

Then set that resource aside and pass her the next one.

Repeat this process until you have gone through all the resources.

If she understands, ask her which resource she likes best. Record her reaction.

Thank her and close the session

**After the interview, discuss your notes and how you thought the young person responded to each resource with your co-interviewer. Did you both interpret the young person’s reaction to each resource similarly? If not, discuss this. Finalise your notes and type them up.**
